# Impact of Activity Tracker Usage in Combination with a Physical Activity Intervention on Physical and Cognitive Parameters in Healthy Adults Aged 60+: A Randomized Controlled Trial

**DOI:** 10.3390/ijerph19073785

**Published:** 2022-03-22

**Authors:** Tina Auerswald, Anna Hendker, Tiara Ratz, Sonia Lippke, Claudia R. Pischke, Manuela Peters, Jochen Meyer, Kai von Holdt, Claudia Voelcker-Rehage

**Affiliations:** 1 Institute of Human Movement Science and Health, Faculty of Behavioral and Social Sciences, Chemnitz University of Technology, 09126 Chemnitz, Germany; tina.auerswald@hsw.tu-chemnitz.de; 2 Department of Neuromotor Behavior and Exercise, Institute of Sport and Exercise Sciences, University of Muenster, 48149 Muenster, Germany; anna.hendker@uni-muenster.de; 3 Department of Psychology & Methods, Jacobs University Bremen GmbH, 28759 Bremen, Germany; t.ratz@jacobs-university.de (T.R.); s.lippke@jacobs-university.de (S.L.); 4 Institute of Medical Sociology, Centre for Health and Society, Medical Faculty, Heinrich Heine University Duesseldorf, 40225 Duesseldorf, Germany; claudiaruth.pischke@med.uni-duesseldorf.de; 5 Leibniz Institute for Prevention Research and Epidemiology–BIPS, 28359 Bremen, Germany; mpeters@leibniz-bips.de; 6 OFFIS–Institute for Information Technology, 26121 Oldenburg, Germany; jochen.meyer@offis.de (J.M.); kai.vonholdt@offis.de (K.v.H.)

**Keywords:** older adults, activity tracker, steps, exercise, cognition, home-based interventions, Simon task

## Abstract

Regular physical activity (PA) is of central importance for healthy aging and has a well-known impact on helping older adults maintain their cognitive and physical health. Thus, we aimed to compare the effectiveness of two physical activity interventions primarily conducted at home (print-based or web-based vs. web-based plus the use of an activity tracker) on cognitive and physical health parameters in older adults. Data of participants (*n* = 551, 60–80 years) were analyzed after being randomly allocated to a waitlist control group (CG), a web-based or print-based intervention group (IG) or a web-based intervention group that also included the use of an activity tracker (AG). Measured parameters were grip strength, endurance (two-minute step test), gait speed (four-meter walk test), cognition (Simon task; balanced integration score (BIS), reaction time and accuracy) and physical self-concept (Physical Self-Description Questionnaire (PSDQ)). We found the highest effect sizes in all measured dimensions for AG (grip strength, endurance, gait speed, reaction time, physical self-concept), followed by IG (endurance, gait speed, reaction time, physical self-concept) and CG (endurance, gait speed, BIS). Findings suggest that a combined web-based and activity tracker intervention may improve physical functions, physical self-concept, and cognition in community-dwelling older adults.

## 1. Introduction

At older ages, cognitive and physical impairments are associated with adverse health outcomes, care dependency and mortality [1,2,3,4,5]. Yet, for older adults, engaging in regular physical activity (PA) can help maintain cognitive and physical health [6,7,8,9,10] and is associated with a decreased risk of functional and cognitive decline [11,12,13,14,15] as well as improved mobility and muscle strength [16,17,18].

At the time of study conception, the World Health Organization (WHO) and the American College of Sports Medicine (ACSM) recommend that adults aged 65 years and older should perform moderate-to-vigorous endurance training for a least 150 min per week (in bouts of at least 10 min) [17,19]. Additionally, older adults (≥65 years) should perform flexibility, strength and balance training two times per week [17,20]. Despite the importance of being physically active, in Germany only 42% of adults aged 65 years and older reach the recommendations for endurance training and only 29% meet the recommendations for strength training [20]. To promote the recommended PA levels and help older adults gain the associated positive physical and cognitive health benefits, interventions targeting the general population of older adults are needed.

Thus far, systematic reviews have suggested that the physical and/or cognitive health of older adults may be effectively promoted by community-based PA exercise programs [21,22,23]. Fitzpatrick and colleagues, for example, investigated the effectiveness of a four-month community-based PA intervention among older adults and found improved physical functions [24]. However, evidence regarding the effectiveness of community-based PA interventions on cognition in older adults is limited [25,26].

In a systematic umbrella review (based on 40 systematic reviews), Olanrewaju and colleagues reported minimal but varying positive effects of planned and structured PA exercise programs on the cognition of older adults [25]. The effectiveness of those programs might be influenced by their delivery mode and tailoring; several studies have indicated that home-based interventions with regular consultations (e.g., telephone interviews; group meetings) are effective in promoting PA in older adults [27,28,29,30,31,32]. Further, the rising use of the internet in older adults in Germany (50% of adults aged 60 years and older use the internet on a regular basis [33]) means that individualized PA programs may be integrated into web-based formats [34]. As such, older populations may be able to utilize home-based interventions that are offered on a print or web basis [35,36,37,38].

As shown by Geraedts and colleagues, exercise programs using web-based techniques seem feasible for frail older adults [38]. Other studies suggest that home-based interventions (e.g., print- or web-based programs) can improve older adults’ physical parameters, such as strength or physical functions [39,40,41,42]. However, further research on how PA interventions conducted at home affect physical parameters is needed to develop recommendations and specify guidelines. Further, findings on whether home-based interventions affect cognitive functioning are limited and inconsistent. While in one study participation in home-based strength and balance training was shown to significantly improve executive functions [43], studies examining the effects of long-term lifestyle interventions have indicated no effects on cognition [44].

Additionally, objective monitoring instruments, such as activity trackers, are becoming increasingly popular and have been used for interventions in both younger and older community-dwelling populations [45,46,47]. Activity trackers might be an effective interventional approach, in that their usage can improve PA behavior [48,49,50]. For example, in a study by Compernolle and colleagues, a combined computer-tailored, pedometer-based PA intervention increased objective and subjective PA levels in working adults (≥18) [51]. In another study, Muellmann and colleagues investigated the benefit of using activity trackers in addition to a website to monitor PA in German older adults; they found that in the group using an activity tracker for three months, compared to the non-tracker group, there were slight, though non-significant, increases in moderate-to-vigorous PA (MVPA) and decreases in participants’ sedentary time [31]. These results suggest that, via increases in PA levels, activity trackers may have an additional impact on health outcomes, such as cognitive and physical function. Indeed, other studies examining the effects of activity tracker interventions on health outcomes in older adults or patients indicate positive effects (e.g., on body mass index (BMI), blood pressure, muscle strength, physical function, LDL cholesterol levels) [45,52,53,54]. However, the impacts of PA interventions conducted at home (e.g., print- or web-based interventions) compared to interventions that also include objective PA monitoring (i.e., via activity trackers) on cognitive and physical function in community-dwelling older adults have not yet been systematically examined. Few studies have investigated the additional effect of activity trackers, and those that have tend to report PA behavior as the main outcome, not physical and cognitive function [31,55]. In sum, evidence about the impact of PA interventions conducted at home (e.g., print- or web-based programs) supported by activity trackers on the cognitive and physical function of older community-dwelling adults is limited.

Thus, the main aim of the current study was to compare the cognitive and physical parameters of older adults living in the Bremen-Oldenburg metropolitan region in Germany for two different PA conditions: those who used print- or web-based interventions and engaged in subjective self-monitoring and those who used web-based interventions and engaged in objective self-monitoring via an activity tracker. Our main research questions were as follows: Does a ten-week PA intervention primarily conducted at home help older adults maintain or even increase their physical and cognitive parameters? Does the use of an activity tracker as an objective feedback device increase the intervention effect even further? Accordingly, the following hypotheses were formulated: (1) Both interventions significantly increase cognitive and physical parameters; and (2) the additional objective feedback provided by wearing an activity tracker further increases the intervention effect.

## 2. Materials and Methods

This study is a subproject of the two PROMOTE studies (PROMOTE I and PROMOTE II), which belong to the larger research network “Physical activity and Health Equity: Primary prevention for healthy aging” (AEQUIPA) funded by the Federal Ministry of Education and Research (BMBF) in Germany. One aim of the network is to develop, implement and evaluate PA interventions to prevent chronic diseases in community-dwelling older adults (60 years or older). PROMOTE, one of the network’s six subprojects, aims to promote a physically active lifestyle; the subproject was conducted in two study phases with similar community-based interventions (PROMOTE I, 2015–2018; PROMOTE II, 2018–2021; [56,57]). Participants of PROMOTE I were randomized to either (1) a web-based intervention, (2) a web-based intervention plus self-monitoring via wearing an activity tracker to track and evaluate their own daily PA, or (3) a waitlist control group (CG). In PROMOTE II, participants were randomized to either (1) a print-based intervention or (2) a web-based intervention, whereby 30% of the web-based intervention group (selected at random) also received an activity tracker. In PROMOTE I and PROMOTE II, participants were free to choose from available time slots during the telephone interview with the study nurse, but were only informed after their decision which group they were assigned to. In both study phases, ten weekly group meetings were offered to all groups except the CG. Study material (e.g., exercise brochures and group meeting manuscripts) are offered in a published toolbox [58]. A detailed description of the study procedure can be found in the published study protocols [56,57].

### 2.1. Participants and Randomization

**Figure 1:**
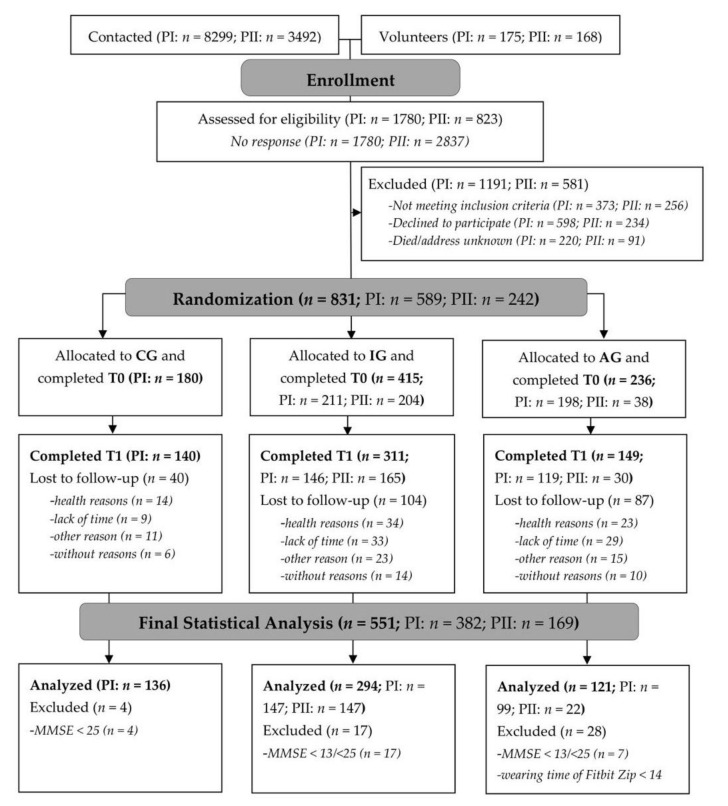
Study flow and randomization procedure of PROMOTE study (PI = PROMOTE I; PII = PROMOTE II). CG = control group; IG = intervention group without activity tracker; AG = activity tracker group receiving the same web-based intervention as the IG.

Within the recruitment process of PROMOTE I and PROMOTE II, a random sample was drawn from the residents’ registration office for communities in the Bremen-Oldenburg metropolitan area; 11,791 persons were contacted and informed about the study and invited to take part (Figure 1). Additionally, participants were recruited via newspaper articles and senior organization institutions. Eligibility for study participation was determined in subsequent telephone interviews with trained study nurses.

The following inclusion criteria were applied for this sub-study: (1) age of at least 60 years; (2) living independently; (3) ability to walk without a walking aid; (4) participation in study assessments and weekly group meetings without external support; (5) basic knowledge of the German language; and (6) the regular availability of a device with internet access. Subjects were excluded if they suffered from a severe illness or planned to go on vacation for more than two weeks during the intervention period. Furthermore, they were excluded if they displayed any sign of cognitive impairment (PROMOTE I: Mini-Mental-State-Examination score of <25/30; PROMOTE II: Mini-Mental-State Examination 2 (brief version) score of <13/15 [59,60]). In PROMOTE I, all participants underwent anthropometric, physical, motor and cognitive tests as well as a self-administered questionnaire. Due to cost issues, in PROMOTE II a random subsample of 114 participants (equally distributed across the intervention groups) underwent anthropometric, physical fitness, and cognitive tests in addition to the self-administered questionnaire, which was collected from all participants. Complete information on the inclusion and exclusion criteria as well as the study procedure can be found in the study protocols for the other studies [56,57].

For this article, data from 551 men and women between 60 and 80 years of age, recruited as a part of PROMOTE I and II, were analyzed (M = 69.38, SD = 4.12). Participants’ data from PROMOTE I and PROMOTE II were pooled, and the following groups were analyzed: a waitlist control group (CG), an intervention group (web-based and/or print-based; IG) or a web-based intervention and activity tracker group (AG), which received the same web-based intervention as the IG plus the activity tracker. Participants of the AG were excluded if they wore the activity tracker for fewer than 14 days. Anthropometric data of all subjects included in this sub-study are presented in Table 1. Baseline assessment for each group took place in pre-scheduled weeks (randomly assigned).

The PROMOTE I study was approved by the Ethics Committee of the Chemnitz University of Technology (TU Chemnitz), Faculty of Behavioral and Social Sciences (number: V-099-17-HS-CVR-PROMOTE-03072015), and was registered at the German Clinical Trials Register (DRKS00010052, date of registration 07-11-2016). For the PROMOTE II study, ethical approval was obtained from the Medical Association in Bremen (RA/RE-635, on 3 July 2018), and the study was registered with the German Clinical Trials Register on 10 January 2019, number DRKS00016073. All study participants were fully informed about the study and provided informed consent.

### 2.2. Measures

#### 2.2.1. Grip Strength (Dynamometer)

In this test we measured grip strength, which is an important indicator of the health of older adults [61]. Grip strength was assessed using a hand grip dynamometer (TKK 5101; Takei Scientific Instruments Co., Ltd., Tokyo, Japan; in kilograms) [62]. The dynamometer was adjusted to the hand size of the participant, the elbow was in a fully extended position, and the arm was not pressed against the body. Participants performed two alternating measurements on each side of the body. Maximal grip strength was quantified as the mean of the highest value out of two trials performed with the right and left hands.

#### 2.2.2. Endurance (Two-Minute Step Test, TMST)

The TMST [63] was used to assess endurance capacity. An adhesive strip was placed on the wall (PROMOTE I), or a rack was assembled (PROMOTE II). Participants stood in front of the wall or rack and raised their thigh to be horizontal with the floor; a mark was placed on the strip or rack at the position corresponding to the middle of the thigh bone, between the kneecap and frontal prominent pelvis bone. For the test, participants stood in front of the wall or rack and lifted their knees in an alternating fashion such that the knees reached the marked location; they alternated the knees as often as they could within two minutes. Before starting the measurement, they were fitted with a chest strap (Polar FS3c; Polar Electro Oy, Kempele, Finland) to measure heart rate (HR), and resting heart rate was recorded in beats per minute (bpm). After finishing the TMST, the researcher recorded the patient’s number of steps (“steps”), as well as their heart rate (HR) directly after trial termination. Thirty seconds after test termination, the HR was recorded again.

#### 2.2.3. Gait Speed (Four-Meter Walk Test)

For measuring gait speed, the four-meter walk test were used [64]. Participants were asked to walk a four-meter distance at their usual speed without shoes. The starting and ending points of the four-meter distance were marked on the floor with lines. For measuring gait speed, subjects started walking 1.5 meters before the start line and stopped a few meters after the end line; the time it took a subject to walk the four meters between the start and end lines was measured in seconds by a handheld stopwatch. In PROMOTE I, the best of two tries was noted with two decimals. In PROMOTE II, only one try was assessed.

#### 2.2.4. Physical Self-Concept (Physical Self-Description Questionnaire, PSDQ)

To evaluate the participants’ physical self-concept, three subscales of the German version of the multidimensional instrument Physical Self-Description Questionnaire (PSDQ) were used; this was part of a self-administered questionnaire [65,66]. For this study we used the subscales for endurance, coordination and strength (Cronbach’s alpha between 0.959 and 0.964). A total of 18 items comprising the subscales endurance, coordination, and strength were judged on a 1–6 (false–true) Likert scale. Afterward, we calculated scores for the categories endurance, coordination and strength, and we calculated a total score.

#### 2.2.5. Cognition (Simon Task)

To test for cognitive capacity, we conducted a Simon task, adapted from the literature [67]. Participants sat in front of a screen where 32 red and blue colored squares were presented on different positions on the screen. Presentation time was 500 ms each, with an inter-stimulus trial of 800–1200 ms. When a blue square emerged, the subject was asked to press the blue key on the keyboard (left side) with their left index finger, whereas when a red square appeared, they responded by pressing the red key (right side of keyboard) with their right index finger. Squares were presented either on the congruent or the incongruent side of the screen. Participants executed 6 blocks of 32 trials with five seconds rest between the blocks (half-time: 20 seconds) and were asked to answer as fast and correctly as possible. The whole measurement procedure lasted about ten minutes.

Outcome measures were the percentage of correct responses (accuracy) and the reaction time (reaction time) across all trials. For all data (reaction time and accuracy of congruent and incongruent trials), means and standard deviations (SD) were calculated. Response times of 300 to 900 ms were defined as valid. Balanced integration scores (BIS) according to Liesefeld et al. (2019) were calculated to integrate speed and accuracy with equal weights for congruent and incongruent trials because the BIS is relatively insensitive to speed–accuracy trade-offs [68]. Pre-processing of the Simon task data was performed using the R 386 3.5.0. package (R Development Core Team).

### 2.3. Procedure

Baseline and follow-up (after 12 weeks) assessments took place in study centers in the Bremen area; here, participants completed the anthropometric, physical, motor and cognitive tests and received a self-administered questionnaire. The AG received a Fitbit Zip (Fitbit, San Francisco, USA) to objectively measure PA. The clip of the Fitbit was attached to the participants’ clothes and recorded their steps taken, distance traveled, and calories burned. Users could easily see their activities by tapping the display. Participants were asked to wear this device as often as possible. Fitbit devices have been validated for use in community-dwelling older adults [69,70,71], and Fitbits have been shown to deliver data that are considerably better than subjective measures, close to that of the “gold standard” of ActiGraph [72]. Participants in the IG and AG received individualized printed or web-based brochures outlining exercises of different difficulty levels and different for men and women; the levels of the exercises were chosen based on their pre-test performance in PROMOTE I, and in PROMOTE II, participants were free to choose which level to perform based on their own fitness. Offered exercises aimed to improve balance, flexibility, strength and endurance, according to the PA recommendations of the WHO [19] and ACSM [17] for moderate-to-vigorous endurance, balance, flexibility, and strength training (for further explanation, please see the study protocols [56,57]). Exercise frequency also aimed to comply with these suggestions (balance and flexibility two times per week, strength on two nonconsecutive days per week, and endurance for at least 150 minutes with moderate-to-vigorous intensity per week in bouts of 10 minutes). All offered exercises were easy to understand and could be executed at home without any equipment or with everyday objects (e.g., a chair, a towel, a wall). Detailed exercise plans can be found in the published toolbox [58].

One week after baseline measures, the first out of ten weekly group meetings (90 min) started, and participants of the IG and AG received access to print- or web-based material for exercise intervention. In the group meetings, which took place in person, different health education topics, e.g., regarding the role of PA for healthy aging and other health-promoting factors (e.g., action strategies, social support) were discussed, and a physical activity session followed. Attendance at the weekly group meetings was strongly recommended but not mandatory. Participants assigned to the delayed intervention CG received no intervention during the first ten-week period but received the intervention of the IG after the post-test, without the weekly group meetings. Twelve weeks after baseline measurement (one week after the end of the ten-week intervention), participants repeated the same assessments for the post-test measurements.

### 2.4. Statistical Analysis

Statistical analyses were conducted with IBM SPSS Statistics for Windows (Version 27.0; Armonk, NY, USA). Repeated measures ANOVA with one within-subject factor (time: pre- and post-test) and one between-subject factor (group: intervention (IG), activity tracker (AG) and control (CG)) were calculated for the physical and cognitive outcome measures (Section 2.2.). Bonferroni-corrected post hoc t-tests were performed to account for multiplicity. *p* values <0.05 were regarded as significant. Effect sizes for repeated measures ANOVAs were reported as partial eta squares and interpreted as small (0.01), medium (0.06) and large (0.14) [73]. Time effects were calculated by paired t-test with effect sizes reported as Cohen’s d and interpreted as small (0.2), medium (0.5) or large (0.8) [73]. Complete case analyses were conducted, i.e., individuals with missing values in pre- or post-measurements were excluded from statistical analyses.

## 3. Results

Descriptive statistics for dependent variables are shown in Table 2.

### 3.1. Grip Strength

**Figure 2:**
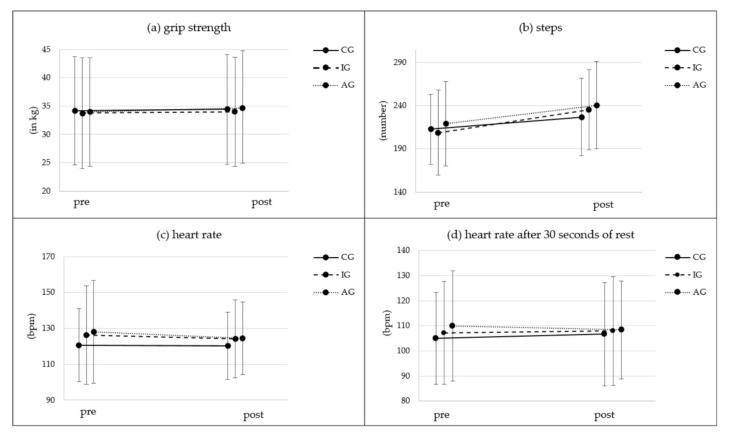
Pre- and post-test results of (**a**) grip strength (kg), (**b**) number of steps per two minutes, (**c**) heart rate (HR) (beats per minute (bpm)) after the two-minute step test and (**d**) HR 30 seconds after two-minute step test (bpm). CG = control group; IG = intervention group without activity tracker; AG = activity tracker group receiving the same web-based intervention as the IG.

Figure 2a shows the grip strength in all three groups. There was a significant time effect for grip strength but no significant group or interaction effect (Table 2). Only small effect sizes were found for time (*ηp^2^* = 0.02). There was a slight descriptive improvement of grip strength in every group from pre- to post-test with only a significant effect for the AG (t(102) = −2.49, *p* = 0.015, d = −245).

### 3.2. Endurance/Cardiovascular Fitness

Figure 2b shows the number of steps participants in each group completed in two minutes as an indicator for endurance capacity. Steps increased significantly in all three groups from pre- to post-test measurement (CG: +14.14 steps; t(134) = −4.90, *p* < 0.001, d = −0.42; IG: +26.41 steps; t(196) = −8.64, *p* < 0.001, d = −0.62; AG: +21.24 steps; t(98) = −6.56, *p* < 0.001, d = −0.66), indicated by a significant time and time x group interaction effect and a large effect size (*ηp^2^* = 0.216) for time (Table 2). Bonferroni-adjusted post hoc analyses revealed no significant difference in the TMST between CG, IG and/or AG. 

In addition to the completed steps, heart rate states were assessed as an indicator of endurance capacity. The measured HR after test termination decreased between time points in the intervention and activity tracker groups (Figure 2c), whereas the HR after 30 seconds of rest decreased between time points just in the activity tracker group (Figure 2d). Heart rate changes, however, were not significantly different between groups or over time and had no significant interaction effects; they just showed small effect sizes (Table 2).

### 3.3. Gait

**Figure 3:**
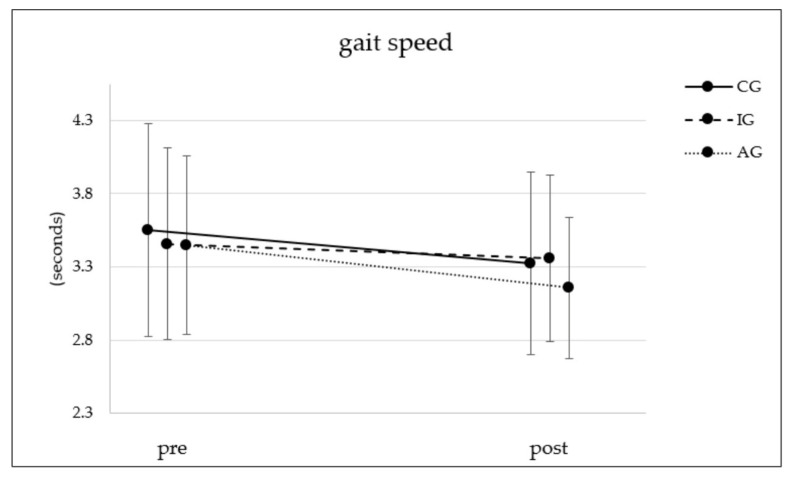
Time (seconds) needed to complete the four-meter walk test. CG = control group; IG = intervention group without activity tracker; AG = activity tracker group receiving the same web-based intervention as the IG.

Figure 3 shows the results of the four-meter walk test for all three groups. All groups improved significantly over time (CG: t(135) = 3.59, *p* < 0.001, d = 0.31; IG: t(200) = 2.13, *p* = 0.035, d = 0.15; AG: t(102) = 4.76, *p* < 0.001, d = 0.47). AG improved the most (−0.29 s), and there was a significant interaction effect between time and group (F(2;437) = 3.13; *p* = 0.045; η² = 0.014) (Table 2). However, Bonferroni-adjusted post hoc analysis revealed no significant difference in the walk test between AG, CG and/or IG.

### 3.4. Physical Self-Concept

**Figure 4:**
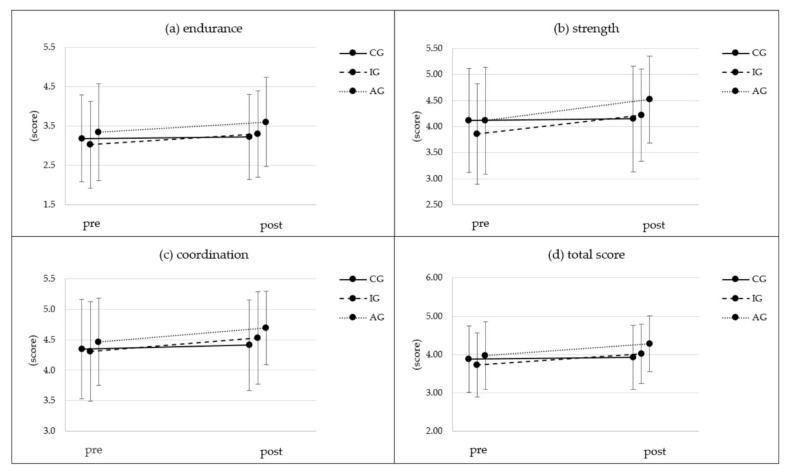
Results for the single dimensions (**a**–**c**) and total score (**d**) of the Physical Self-Description Questionnaire pre- and post-intervention for all three groups. CG = control group; IG = intervention group without activity tracker; AG = activity tracker group receiving the same web-based intervention as the IG.

Figure 4 shows the results for participants’ physical self-concept in all three groups. Endurance, coordination and strength scores showed significant time effects and time x group interactions, with an additionally significant group effect for endurance and strength and a trend for coordination. Effect sizes for time and the time x group interaction were small for endurance and coordination, whereas strength showed a large time effect size (*ηp^2^* = 0.152) and a medium time x group interaction effect size (*ηp^2^* = 0.061). Endurance, coordination and strength improved significantly in IG and AG between pre- and post-test measurements (Table 3).

The overall PSDQ score also showed improvements in all groups, with significant results for group and time x group and a large effect for time (*ηp^2^* = 0.152; Table 2). IG and AG showed significant improvements in overall PSDQ scores between pre- and post-test measurements (Table 3). Bonferroni-adjusted post hoc analysis revealed significant time differences in strength (*p* = 0.017; 0.28; 95%-CI (0.037; 0.518)) and overall score (*p* = 0.012; 0.25; 95%-CI (0.044; 0.461)) for groups AG and IG.

### 3.5. Cognition

**Figure 5:**
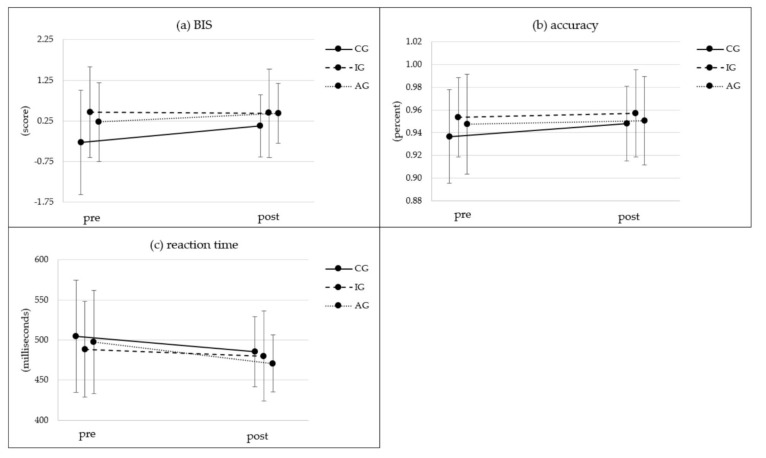
Balanced integration score for the Simon task: (**a**) BIS, (**b**) accuracy (%) and (**c**) reaction time (ms) in pre- and post-test conditions. BIS = balanced integration score; CG = control group; IG = intervention group without activity tracker; AG = activity tracker group receiving the same web-based intervention as the IG.

Figure 5 shows the results of the Simon task in all three groups. Overall, the balanced integration score (BIS) indicated a significant time effect with significant improvements in the CG (t(29) = −2.07, *p* = 0.047, d = −0.38) and descriptive improvements in the AG but not for the IG, which started at a higher performance level. There were no significant differences between groups and only a trend (*p* = 0.052) for the group x time interaction (Table 2). Bonferroni-adjusted post hoc analysis revealed no significant differences in BIS score between CG, IG and/or AG.

Looking at the accuracy and the response reaction times separately (Figure 5b,c), we found only a significant time effect but no group or interaction effects. All three groups reduced their reaction times and increased the accuracy of their responses (Table 2), with significant improvements of reaction time in IG (t(87) = 2.15, *p* = 0.034, d = 0.23) and AG (t(23) = 2.89, *p* = 0.008, d = 0.59). Participants in the AG improved their reaction time by 26.80 ms, while the CG (19.04 ms) and IG (8.38 ms) improved less. Effect sizes in reaction time were medium to large (*ηp^2^* = 0.122) for the time effect; all other effects were just small. There were no significant improvements in accuracy for CG, IG or AG between pre- and post-test measurements.

## 4. Discussion

The current study investigated the effects of two PA interventions primarily conducted at home (a print- or web-based intervention with only subjective self-monitoring vs. a web-based intervention with additional objective monitoring via an activity tracker) on cognitive and physical parameters in older German adults (compared to a delayed intervention control group). Results of the present study suggest that a combination of web-based and activity tracker PA interventions may improve physical function (strength, endurance, gait speed) and physical self-concept and may partially improve cognitive function in community-dwelling older adults.

### 4.1. Impact of PA Intervention Primarily Conducted at Home (With or without Additional Use of an Activity Tracker) on Physical Parameters

Our results showed significant improvements in grip strength, an indicator of functional health, after ten weeks of intervention for the activity tracker group only, whereas the control group and intervention group that did not use a tracker showed no significant improvements in grip strength. This finding suggests that combining a primarily home-based PA intervention with wearing an activity tracker may positively influence older adults’ strength. The result is in line with earlier studies on pre-frail and frail as well as healthy older adults. In pre-frail and frail older adults, a home-based physical and nutritional intervention increased participants’ grip strength [41,74]. In another study, Takahashi and colleagues found that the grip strength of adults significantly improved from wearing activity trackers and receiving motivational feedback (mean age 63.4 yrs.) [75]. Similarly, Talbot and colleagues showed that isometric strength increased by 21% in community-dwelling older adults wearing an activity tracker, while it decreased by 3.5% in the control group (arthritis self-management education program) [54].

However, research investigating the effect of a PA intervention combined with wearing activity trackers on the grip strength of healthy older adults is rather sparse and inconsistent. Snyder and colleagues, for example, did not find an improvement in grip strength for older adults using pedometers [53]. Hand grip strength has been shown to be a valid indicator of muscle strength, mortality, quality of life, and heart health [76,77]. Thus, our results suggest that becoming more or being sufficiently physically active might contribute improved grip strength, thereby potentially affecting these factors.

Endurance capacity was measured by the TMST and was assessed as the number of steps completed in two minutes and the participant’s heart rate 30 seconds after trial termination. We found a significant group x time interaction for the number of steps, indicating that changes in endurance capacity were higher for both intervention groups than for the control group: IG (+26.41 steps) and AG (+21.24 steps) descriptively showed the greatest improvements in steps between pre- and post-test measurements compared to the CG (+14.14 steps). Follow-up tests, however, revealed that steps increased significantly in all three groups from pre- to post-test measurement. In a different study, Hong and colleagues investigated the effect of a tele-exercise intervention with real-time interactions on sarcopenia-related factors of body composition and functional fitness among community-dwelling older adults; they found no significant effects for the TMST. However, the authors found positive effects on total-body skeletal muscle mass, appendicular lean soft tissue, lower limb muscle mass and chair sit-and-reach scores [78]. Heart rate 30 seconds after trial termination also did not differ between groups, but it did decrease descriptively only in the activity tracker group.

Thus, results of the current study show a tendency for improvements in endurance capacity of community-dwelling older adults after ten weeks of a print- or web-based intervention with or without additional use of activity trackers. A meta-analysis by Beishuizen and colleagues concluded that web-based interventions, with or without using activity trackers, have the potential to improve older persons’ cardiovascular risk factors, such as blood pressure and weight. However, studies have revealed only modest effects and a decline of effects with time [79]. One study included in the meta-analysis showed that the combination of a pedometer and an interactive web-based intervention improved the vascular endothelial function, compared to a control group and a pedometer-usage only group [80]. Similarly, a systematic review and meta-analysis by Franssen and colleagues showed that consumer wearable activity trackers have beneficial effects on cardio metabolic health of adults with chronic diseases [52]. Although the results of the current study cannot be compared with previous research due to the intervention type and outcomes, the present evidence suggests that a combination of a primarily home-based intervention (especially web-based intervention) and objective monitoring might have a positive impact on endurance capacity.

Various studies have shown that home-based interventions can improve physical function, including gait speed in older adults [39,81,82,83,84], while evidence on the association between the use of activity trackers and gait speed is limited. To date, the few studies examining this issue have indicated that wearing activity trackers can improve gait speed in older adults [53,54]. We also found a significant time x group interaction. Although all three groups showed significant improvements in four-meter walking time between pre- and post-test measurements, the AG improved the most (−29 seconds), suggesting a positive tendency of monitoring devices to improve gait speed, in line with previous research.

One’s physical self-concept is an important mediator in physical activity, as well as a valuable outcome in itself [85]. We assessed three subscales of the PSDQ related to self-described endurance, coordination and strength competence. Our results showed significant time x group interactions for all dimensions of the PSDQ, with large time effect sizes for subjective strength and the PSDQ overall score. Scores on all three subscales (endurance, coordination, strength) and the overall score improved significantly between pre- and post-test measurements in IG and AG, suggesting that PA interventions primarily conducted at home with or without additional usage of activity trackers increases the subjective perception of physical performance. Previous studies on this specific topic are limited and inconsistent. Griffith and colleagues showed that a home-based walking intervention improved self-reported physical function in cancer patients (mean age: 60.2 years) [86].

In contrast, Matson and colleagues did not show significant improvements in self-reported health outcomes in older adults who completed a sedentary behavior intervention and used activity trackers [87]. Conde-Pipó and colleagues found that practicing sport activities for more than 150 min/week was associated with higher scores of physical self-concept in middle-aged and older adults [88]. Other studies with young adults have shown that changes in physical self-concept through participating in physical activity can lead to improvements in well-being [85,89,90]. Presumably, we might have induced similar effects by our study.

In summary, our results indicate that home-based PA interventions conducted primarily at home can improve objective and subjective physical performance. In particular, the combination of a home-based intervention and the additional use of an activity tracker seems to positively influence physical parameters, such as strength endurance and walking. Results are in line with previous research findings. However, research about this topic in community-dwelling older adults is sparse, and no studies with a comparable intervention currently exist. Thus, the findings of this study need to be replicated, and mechanisms that explain the effects are still needed.

### 4.2. Impact of PA Intervention Primarily Conducted at Home (With or without Additional Use of an Activity Tracker) on Cognitive Parameters

Our results revealed only a trend for the time x group interaction effect regarding the balanced integration score and no effects for reaction time or accuracy. Against our assumption, the control group showed significant improvements in balanced integration score over time, while AG improved only descriptively (+0.21), and IG did not improve. However, IG and AG started at a higher performance level, which may have been interrelated with the results in terms of a ceiling effect. Despite the missing interaction effect for reaction time, IG and AG improved significantly in reaction time, with AG improving descriptively the most (−26.8 ms). The results indicate that for cognitive tasks, the PA intervention conducted primarily at home with or without using an activity tracker positively affected reaction time but not accuracy.

While previous research has suggested that structured PA programs (i.e., group delivered, center-based) can positively influence cognitive parameters [25], evidence on the impact of home-based interventions and/or the use of activity trackers on cognition is sparse. Scherder and colleagues showed that an individual treatment (walking and hand/face exercises) nearly significantly improved executive function in older adults with mild cognitive impairments [91]. Lautenschlager and colleagues showed modest improvements in cognitive parameters after six months of a home-based intervention for older adults with subjective memory impairments [92]. Van Gelder and colleagues suggested that walking can improve cognitive function in elderly people without cognitive decline [93], indicating that interventions aiming to promote walking positively influence cognitive parameters; such interventions include wearing activity trackers with motivational feedback [31,48,51]. Further research about the impact of mostly home-based PA and/or activity tracker interventions on cognitive parameters in (healthy) older adults is needed to underline previous findings as well as the results of the present study and to provide recommendations for further interventions.

### 4.3. Strengths and Limitations

A major strength of the study was that we investigated the effects of two different types of interventions on objective parameters of cognitive function and objective and subjective parameters of physical function for community-dwelling older adults, as compared to a control group in a randomized intervention trial. Thus far, few studies have investigated the influence of primarily home-based PA and/or activity tracker interventions on cognition and physical parameters in general and by using objective measures. Existing studies have often targeted younger populations. In addition, cognitive and physical variables were often investigated in separate studies, unlike in this study where we investigated them in a combined way.

Another strength of the study arose from using a combination of two project phases for data analysis (PROMOTE I and PROMOTE II), as this provided a high sample size (*n* = 551) and enabled extensive analysis of physical activity interventions across multiple time periods and modes of delivery. However, combining the two projects also involves several limitations. PROMOTE I and PROMOTE II are similar in design, but differences exist that might have affected the results. Both studies took place at different points in time, such that various factors (e.g., weather influences) could have affected the data’s comparability [94]. In addition, the study designs differed in some respects. First, only PROMOTE I included a delayed intervention control group, which accounts for the relatively small number of participants in the CG. Furthermore, PROMOTE I distinguished (1) web-based interventions from (2) web-based interventions plus the use of activity trackers, while PROMOTE II additionally implemented a purely print-based intervention. To combine the outcomes, we pooled the data for participants receiving the print-based and web-based interventions as well as data for the two web-based plus activity tracker interventions of PROMOTE I and II. We were, therefore, not able to draw any conclusions on the differences between print-based or web-based interventions for the investigated parameters separately. However, studies have shown that web-based and print-based physical activity interventions can increase physical activity in middle-aged and older adults similarly [95,96], suggesting few differences in impact. The recruitment strategy of PROMOTE I and II (mainly targeted active, healthy and science-interested community-dwelling older adults) as well as the systematic dropout in PROMOTE I might also limit the analyzed sample’s representativeness of community-dwelling older adults and should be considered when interpreting the results [97,98]. The recruitment strategy was adapted from PROMOTE I to II because of a physically active and highly educated sample. High dropout rates were particularly prevalent in the AG (36.9%) between pre- and post-test measurements, suggesting that older participants felt incapable of dealing with websites and/or activity trackers such as the Fitbit Zip [31]. Current evidence suggests that activity tracker devices are usable and accepted by older adults, even frail older populations [48,99,100]. However, future research should more strongly focus on adapting activity trackers to the needs of the target groups. For example, more easy-to-handle devices, such as wristbands, should be considered, especially for individuals with limitations with vision, fine motor skills or technology affinity [101].

## 5. Conclusions

The results of the present study indicate that for community-dwelling older adults, participating in a PA intervention primarily conducted at home with additional use of an activity tracker may positively affect certain parameters of physical and cognitive health. We found effects for all dimensions except strength. Thus, the hypotheses of our study can be partially accepted, and the potential of activity trackers was demonstrated with selected target parameters. Moreover, studies have demonstrated that activity trackers can be used and accepted by older populations [48,99,100]. Thus, considering the growing use of the internet and digital devices, even in aging populations, home-based interventions in combination with objective motivational feedback (i.e., via activity trackers) offer the possibility of increasing physical activity and its associated positive effects on health, such as physical and cognitive parameters, in a cost-effective way. These findings contribute to further development of specific recommendations for physical activity interventions for community-dwelling older adults.

## Figures and Tables

**Table 1:** Anthropometric data (mean = M and standard deviation = SD) of the participants recorded by self-report via questionnaire. CG = control group; IG = intervention group without activity tracker; AG = activity tracker group receiving the same web-based intervention as the IG.

	CG	IG	AG
N	136	294	121
Gender			
female (*n*, %)	71, 52.2%	183, 62.2%	66, 54.5%
male (*n*, %)	61, 44.9%	109, 37.1%	55, 45.5%
Missing (*n*, %)	4, 2.9%	2, 0.7%	0, 0.0%
age (years) (M ± SD)	70.22 ± 3.02	68.71 ± 4.47	70.08 ± 4.02
height (cm) (M ± SD)	169.31 ± 8.72	169.88 ± 8.59	170.40 ± 8.48
weight (kg) (M ± SD)	80.23 ± 14.50	78.99 ± 14.04	78.60 ± 15.28
BMI (kg/(height in m)^2^) (M ± SD)	27.96 ± 4.57	27.36 ± 4.37	26.99 ± 4.40

**Table 2:** Mean values (M), standard deviations (SD) and results of ANOVA for evaluated parameters of dependent variables separated by group (control group (CG), intervention group (IG) and activity tracker group (AG)). T0 = pre-test; T1 = post-test 12 weeks after baseline.

															Inner Subject Contrasts					Between Subject Contrasts		
Dimension	Parameter	CG				IG				AG				Time			Time * Group				Group	
		*n*	M	SD		*n*	M	SD		*n*	M	SD		F-value	*p*-value	*ηp^2^*	F-value	*p*-value	*ηp^2^*	F-value	*p*-value	*ηp^2^*
Grip strength [dynamometer]	T0 [kg]	134	34.18	9.61		198	33.76	9.82		103	34.03	9.49	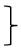 *	(1, 432) = 8.88	0.003 *	0.020	(2, 432) = 1.06	0.349	0.005	(2, 432) = 0.12	0.888	0.001
	T1 [kg]	134	34.42	9.68		198	33.99	9.68		103	34.69	10.10										
Endurance [two minute step test]	steps T0 [#]	135	212.61	40.52	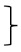 **	197	208.80	49.41	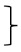 **	99	219.11	49.17	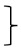 **	(1, 428) = 117.90	<0.001 **	0.216	(2, 428) = 4.32	0.015 *	0.02	(2, 428) = 1.68	0.188	0.008
	steps T1 [#]	135	226.75	44.97		197	235.21	46.62		99	240.35	50.64										
	heart rate T0 [bpm]	121	120.57	20.46		186	126.24	27.58		96	128.01	28.77		(1, 400) = 2.74	0.099	0.007	(2, 400) = 0.58	0.563	0.003	(2, 400) = 2.74	0.066	0.013
	heart rate T1 [bpm]	121	120.31	18.88		186	124.19	21.81		96	124.36	20.32										
	heart rate post T0 [bpm]	119	105.03	18.30		186	107.16	20.59		96	109.95	22.14		(1, 398) = 0.09	0.759	0.000	(2, 398) = 0.93	0.396	0.005	(2, 398) = 0.85	0.427	0.004
	heart rate post T1 [bpm]	119	106.65	20.56		186	107.96	21.74		96	108.38	19.54										
Gait [four meter walk test]	T0 [seconds]	136	3.50	0.73	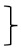 **	201	3.41	0.66	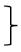 *	103	3.40	0.62	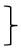 **	(1, 437) = 37.73	<0.001 **	0.079	(2, 437) = 3.13	0.045 *	0.014	(2, 437) = 2.10	0.124	0.010
	T1 [seconds]	136	3.27	0.63		201	3.31	0.57		103	3.11	0.48										
Physical self-concept [PSDQ]	Score T0 [mean]	126	3.88	0.87		282	3.73	0.83	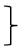 **	115	3.97	0.88	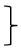 **	(1, 520) = 92.90	<0.001 **	0.152	(2, 520) = 1.,03	<0.001 **	0.048	(2, 520) = 4.35	0.013 *	0.016
	Score T1 [mean]	126	3.93	0.84		282	4.01	0.78		115	4.27	0.73										
	Endurance T0 [score]	130	3.18	1.11		285	3.03	1.10	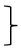 **	116	3.34	1.24	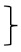 **	(1, 528) = 39.87	<0.001 **	0.070	(2, 528) = 6.01	0.003 *	0.022	(2, 528) = 3.55	0.029 *	0.013
	Endurance T1 [score]	130	3.23	1.09		285	3.30	1.10		116	3.60	1.14										
	Coordination T0 [score]	129	4.35	0.82		285	4.31	0.82	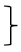 **	116	4.47	0.72	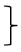 **	(1, 527) = 46.47	<0.001 **	0.081	( 2, 527) = 4.27	0.014 *	0.016	(2, 527) = 1.81	0.061	0.011
	Coordination T1 [score]	129	4.41	0.75		285	4.53	0.76		116	4.69	0.61										
	Strength T0 [score]	126	4.12	1.00		282	3.86	0.97	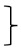 **	115	4.11	1.03	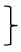 **	(1, 520) = 93.23	<0.001 **	0.152	(2, 520) = 16.98	<0.001 **	0.061	(2, 520) = 3.85	0.022 *	0.015
	Strength T1 [score]	126	4.15	1.02		282	4.22	0.89		115	4.52	0.84										
Cognition [Simon Task]	BIS^1^ T0	30	−0.21	1.27	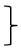 *	88	0.46	1.12		24	0.22	1.00		(1, 139) = 5.79	0.017 *	0.040	(2, 139) = 3.02	0.052	0.042	(2, 139) = 2.70	0.07	0.037
	BIS^1^ T1	30	0.13	0.78		88	0.44	1.10		24	0.43	0.75										
	Reaction time T0 [ms]	30	504.55	71.20		88	488.38	59.97	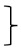 *	24	497.61	65.72	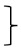 *	(1, 139) = 19.31	<0.001 **	0.122	(2, 139) = 2.12	0.124	0.030	(2, 139) = 0.48	0.619	0.007
	Reaction time T1 [ms]	30	485.51	44.37		88	479.99	56.69		24	470.81	36.35										
	Accuracy T0 [%]	30	93.67	0.04		88	95.36	0.04		24	94.75	0.05		(1, 139) = 5.22	0.024 *	0.036	(2, 139) = 1.14	0.322	0.016	(2, 139) = 1.53	0.221	0.022
	Accuracy T1 [%]	30	94.80	0.03		88	95.69	0.04		24	95.04	0.04										

* p<0.05; ** *p*<0.001; ^1^ BIS=Balanced integration score.

**Table 3:** Paired sample t-test for time effect of PSDQ subtests and overall score between pre- and post-test measurements. CG = control group; IG = intervention group without activity tracker; AG = activity tracker group receiving the same web-based intervention as the IG. Negative values indicate a lower value at pre- than at post-test measurement.

Group	Parameter (pre * post)	t	Df	*p*-Value	Cohen’s d
CG	PSDQ Endurance	0.688	129	0.493	−0.060
	PSDQ Coordination	−1.346	128	0.181	−0.199
	PSDQ Strength	−0.621	125	0.536	−0.055
	PSDQ Overall Score	−1.154	125	0.251	−0.103
IG	PSDQ Endurance	−7.184	284	<0.001 *	−0.426
	PSDQ Coordination	−6.862	284	<0.001 *	−0.406
	PSDQ Strength	−10.288	281	<0.001 *	−0.613
	PSDQ Overall Score	−10.245	281	<0.001 *	−0.610
AG	PSDQ Endurance	−4.639	115	<0.001 *	−0.431
	PSDQ Coordination	−4.961	115	<0.001 *	−0.461
	PSDQ Strength	−7.355	114	<0.001 *	−0.686
	PSDQ Overall Score	−7.422	114	<0.001 *	−0.692

* *p* < 0.001.

## Data Availability

The data presented in this study are available on request from the corresponding author. The data are not publicly available due to privacy and ethical restrictions.
